# A randomised controlled feasibility trial for an educational school-based mental health intervention: study protocol

**DOI:** 10.1186/1471-244X-12-23

**Published:** 2012-03-22

**Authors:** Katharine Elizabeth Chisholm, Paul Patterson, Carole Torgerson, Erin Turner, Max Birchwood

**Affiliations:** 1 School of Psychology, University of Birmingham, Edgbaston, Birmingham B15 2TT, UK; 2 CLAHRC Public Health Team, Research & Innovation, 68 Hagley Road, Birmingham B16 8PF, UK; 3 School of Education, University of Birmingham, Edgbaston, Birmingham B15 2TT, UK; 4 Early Intervention Services, Birmingham and Solihull Mental Health Foundation Trust, Newington Resource Centre, Newington Road, Marston Green, Birmingham B37 7RW, UK

**Keywords:** Adolescence, Mental health, Stigma, Contact, School, Intervention

## Abstract

**Background:**

With the burden of mental illness estimated to be costing the English economy alone around £22.5 billion a year [1], coupled with growing evidence that many mental disorders have their origins in adolescence, there is increasing pressure for schools to address the emotional well-being of their students, alongside the stigma and discrimination of mental illness. A number of prior educational interventions have been developed and evaluated for this purpose, but inconsistency of findings, reporting standards, and methodologies have led the majority of reviewers to conclude that the evidence for the efficacy of these programmes remains inconclusive.

**Methods/Design:**

A cluster randomised controlled trial design has been employed to enable a feasibility study of 'SchoolSpace', an intervention in 7 UK secondary schools addressing stigma of mental illness, mental health literacy, and promotion of mental health. A central aspect of the intervention involves students in the experimental condition interacting with a young person with lived experience of mental illness, a stigma reducing technique designed to facilitate students' engagement in the project. The primary outcome is the level of stigma related to mental illness. Secondary outcomes include mental health literacy, resilience to mental illness, and emotional well-being. Outcomes will be measured pre and post intervention, as well as at 6 month follow-up.

**Discussion:**

The proposed intervention presents the potential for increased engagement due to its combination of education and contact with a young person with lived experience of mental illness. Contact as a technique to reduce discrimination has been evaluated previously in research with adults, but has been employed in only a minority of research trials investigating the impact on youth. Prior to this study, the effect of contact on mental health literacy, resilience, and emotional well-being has not been evaluated to the authors' knowledge. If efficacious the intervention could provide a reliable and cost-effective method to reduce stigma in young people, whilst increasing mental health literacy, and emotional well-being.

**Trial registration:**

ISRCTN: ISRCTN07406026

## Background

There is growing pressure on schools in England to address stigma and misconceptions of mental illness [2-5] as well as increasing emotional well-being [6,7] in addition to traditional academic curricula. Currently there is little agreement as to what approach might most successfully address these issues [9-11].

Over half of all lifetime mental disorders have their onset during childhood and adolescence [12] yet young people's knowledge of mental illness is often incomplete [13] or influenced by sensationalised reports in the media [14]. Developmentally, adolescence is a complex period, with the majority of young people experiencing some level of distress or emotional problems. The adolescent brain has been shown to confer higher risk for mental illness due to a difference in the maturation of the developing brain and the adolescent emotional and cognitive systems [15]. Adolescents also experience high levels of schizotypal thinking [16], something which among adults is related to high vulnerability to mental illness [17].

Individuals with mental illness experience discriminatory attitudes in almost all areas of their lives [18,19], and stigma, alongside poor mental health literacy, can increase barriers to help-seeking [20,21]. Prevalence rates for psychotic disorders such as schizophrenia increase rapidly between the ages of 15 to 17 [12] with children and adolescents who experience early onset anxiety disorders such as phobias and separation anxiety disorder more likely to develop co-morbid disorders of substance use, later-onset anxiety, or mood [12]. Earlier age of onset for many mental illnesses is also correlated with greater severity [22], persistence [23] and increased resistance to treatment [24].

Young people themselves report that they would like to receive more information regarding mental health. Both Kidger et al. [25] and Woolfson et al. [26] found that students stated that they were dissatisfied with the mental health education they were receiving. Olsson and Kennedy [13] found that less than 30% of students recalled any discussion of mental health during school lessons, despite the fact that mental health education was mandatory in the area their research was conducted.

There is growing evidence to suggest that early interventions designed to optimise well-being or reduce mental illness and initiated during childhood and adolescence may help reduce mental health problems in later life [27-29]. The UK National Healthy Schools Programme attempts to address this, and advocates increasing emotional health and well-being (EHWB) support for students [30]. Schools engaged in this programme are expected to address subjects related to social skills and emotional health to their curriculum with targets including 'combating stigma and discrimination'; and 'encouraging the participation of children and young people in activities designed to build their confidence and self esteem' to achieve National Healthy Schools Status. There is however, no obligation for schools to take part and some remain uninvolved. Additionally, with lack of monitoring and threat of further funding cuts, the current programme appears vulnerable.

### Previous interventions

Reducing stigma and discrimination of mental illness, improving mental health literacy, reducing a particular mental illness, or promoting resilience are all popular methods for tackling mental health issues in schools [31-40]. Such interventions often demonstrate significant benefits such as reducing stigma, conduct problems and increasing prosocial behaviour [41], challenging negative opinions of psychosis [42], and can have a positive impact on interrelated factors such as school achievement, school climate, and reduced emotional behavioural and disciplinary problems [43-45]. The abundance of interventions are however, not easily comparable, and identifying which components of an intervention are successful, and which components lack utility, is often not possible. This has led to some large-scale interventions being instigated without a clear understanding of the likely efficacy of trial components. For example the *BeyondBlue *[46] trial in Australia included 50 schools in a five year intervention aimed at reducing and preventing depression and reported flat results [47]. Feasibility trialling and careful evaluation is needed to capture the potential problems as well as benefits of school interventions prior to the widespread dissemination of larger scale projects [9,48,49].

Many systematic reviews now concur that whole school approaches are the most effective model and emphasise the need to promote mental health universally rather than solely targeting the reduction of mental illness [11,50,51]. A greater investment in long-term approaches are also favoured by reviewers [11,52] and it has been suggested that future research should include more detailed analysis of demographic variables such as ethnicity and gender [9].

Regarding stigma and mental health literacy, fewer systematic reviews have been conducted into school-based studies. Kelly et al.'s [21] review concluded that for adolescents, good mental health literacy is associated with more positive outcomes for emotional well-being but add a caveat that very few school interventions to improve mental health literacy have been evaluated and even fewer *well *evaluated. Pinfold et al.'s [53] systematic review suggested that contact with individuals who describe personal experience of mental illness was more likely to lead to reductions in reported stigma; in contrast, a systematic review by Schachter et al. [48] failed to find any 'even remotely ideal investigation[s] whose results regarding possible benefits we can confidently accept as being reliable and valid' (p. 22). Schachter et al. criticised studies for a range of reasons including; inadequate study descriptions; poor research methodology; inadequate interventions; and failing to measure or control for relevant baseline characteristics of participants. Funding is also an issue in the present climate, with programmes designed to impact upon mental health issues in schools needing to demonstrate value for money as well as reliable and consistent effect sizes [9,54]. In summary, further well-designed research in these fields is warranted.

### Contact and stigma

Corrigan et al. [55] identify three strategies for challenging stigmatising attitudes and prejudice towards mental illness: 'protest', 'education', and 'contact'. 'Protest' involves condemning prejudicial attitudes; if a company were employing an advertising campaign that was deemed to be stigmatising, public protest may encourage changing of the advert, thus suppressing the stigmatising attitude. 'Education' seeks to encourage an increase in knowledge and awareness regarding mental illness, thereby dispelling myths in the belief that the more individuals understand the less prejudiced they will be. 'Contact' reasons that the more contact an individual has with someone who has experienced mental illness, the less stigmatising they will be, although the relationship is correlational - it may equally be the case that those who hold less stigmatising attitudes towards mental illness are happier to have contact with individuals experiencing mental illness [55]. Rusch et al. [56]note that if contact is employed to facilitate attitude change then this must be carried out under a controlled set of circumstances if the best possible outcome is to be achieved, otherwise the intervention may have a negative rather than positive outcome. Allport [57] who first proposed the contact hypothesis, similarly claimed that contact needed to involve individuals of equal status with the 'in' and 'out' groups working together rather than in competition with one another. Of protest, education, and contact, Corrigan et al. argue that contact is the most effective, followed by education, with protest having little significant impact on participants. Like Minds [19] add that contact married to education is a powerful combination for combating stigma.

Contact has been employed as a strategy to reduce stigma and increase understanding of mental health issues in a number of studies with adults [55,58] but rarely in school interventions [53,56]. Results have however shown promise, and contact may be a way of increasing the impact of interventions by facilitating engagement and overcoming barriers to learning. To date, contact has not been utilised to improve mental health literacy and emotional well-being in young people, but there is some evidence to suggest that it might have a favourable effect as two recent qualitative studies have indicated that young people wish to be taught about emotional well-being and mental health issues by someone who has lived experience of mental illness [25,26].

### The current study

The SchoolSpace intervention programme has been designed to measure the impact of an educational intervention on three core targets: reduction in stigma related to mental illness; mental health literacy; and promotion of mental health, with reduction in stigma related to mental illness being the primary outcome. Previous research has suggested that these may be interlinked [41]. For example, someone who doesn't *understand*, or is *prejudiced *against mental illness, may not seek help [20,21], leading them to be less *resilient*. The primary research question asks; 'is education in combination with contact better than education alone for reducing stigma?' Based on previous research [26,53] it is hypothesised that participants in the contact and education condition will report significantly reduced stigma towards mental illness compared with participants in the education only (control) condition.

Secondary research questions relate to how contact in combination with education may influence participants' a) mental health literacy, and b) emotional well-being and resilience, compared to education alone. In addition to this the trial will assess any influence of contact on attitudes to help-seeking. Previous research has found that stigma is a major barrier to help-seeking [20], and that low mental health literacy can lead to a delay in help-seeking [34]. It is therefore hypothesised that a reduction in stigma and increased mental health literacy will be associated with an increase in positive attitudes towards help-seeking.

Additionally, schizotypal thinking, which is linked to vulnerability to mental illness [17] and is common during adolescence [16] will also be measured. This addresses whether higher rates of schizotypal thinking in adolescence are linked to adolescents' increased vulnerability to developing mental illness compared to other age groups, or if such thinking patterns are developmental in some way [59]. If they are a sign of an adolescents increased vulnerability to mental illness then with increased resilience and increased mental health and emotional well-being, we hypothesise that rates of schizotypal thinking should reduce in participants. However, if schizotypal thinking is developmental than we might expect to see the opposite trend, with schizotypal thinking unaffected by any changes in mental well-being or resilience.

## Method/Design

SchoolSpace is a randomised controlled trial with two conditions: an experimental condition which includes contact with a young person with lived experience of mental illness as well as education, and an active control condition, which includes education but no contact. Measures will be taken 3 weeks prior to the intervention, and at both 2 weeks and 6 months post intervention. Random allocation will take place at the level of school and blocking will be used to randomly stratify classes to different conditions. Random allocation will be independent and concealed. Randomisation will be undertaken by an individual independent of the research team, and will take place after pre-test. The intervention has been designed in accordance with CONSORT guidelines [60,61].

### Participants

7 schools will take part in the intervention, with students aged 12-13. Schools will be chosen to represent a cross section of demographics, including different ethnic groups and genders. The average year group within Birmingham secondary schools takes approximately 150 students enabling up to 1050 participants in this feasibility trial.

### Ethical approval

The study was granted ethical approval by The University of Birmingham ethics committee in June 2010 (reference number ERN_10-0397), and the primary author is funded through the NIHR CLAHRC (Collaborations for Leadership in Applied Health Research and Care) Birmingham & Black Country programme.

### Development and piloting of the intervention

Original intervention materials were developed by K.C., E.T. and P.P. in collaboration with teachers and service-users with additional educational resources evolved from the work of Dr Gary O'Reilly [62] and the Staffordshire Changes YP mental health programme [63]. Focus groups were also conducted with young people by K.C. to aid the development of the programme. Interventions tend to be more successful if they are delivered over time, rather than in one intensive session [9], however the current intervention is a feasibility trial, and due to the constraints of resources, it was decided that a one-day intervention would be used, with the expectation that the intervention will be developed into a longer term intervention plan for evaluation in the future, should it prove to be successful.

The intervention was piloted in one school to assess the practicality; timings, level of pitch, and suitability of materials for the target age group. Classes were randomised to the Education and Contact condition and Education Only conditions. Procedure of the pilot intervention is identical to the feasibility trial.

### Feasibility trial

The feasibility trial intervention covers three interrelated subjects; 1. Stigma of mental illness, 2. Mental health literacy, and 3. Improving our own mental health. Pupils receive four hours aimed to impact upon the former two of these subjects and two hours aimed to impact upon the latter. In the education and contact condition a young person with experience living with mental illness will work with the students as one of the teaching assistants and will lead a 10-20 minute presentation and interactive discussion about living with a mental illness.

The intervention will be delivered by NHS mental health specialist staff and service-users. Training and workshop notes are provided for all individuals prior to their facilitating the interventions to ensure fidelity of implementation. The intervention will be delivered to students in their normal class size and environment.

### Procedure

#### Stage 1; consent and pre-test

Schools in the Birmingham area will be approached and invited to take part in the research, drawn from the full range of socio-economic and socio-cultural settings. Once a school has agreed to participate, consent letters will be sent out to the parents of all participating students.

Pre-intervention questionnaires will be completed 3-4 weeks prior to the intervention day. A self-generated code will be used to match participant answers over time whilst preserving anonymity. This code has a reported 92% success rate [64]. It must therefore be expected that a minimum of 8% of participants may be lost over time due to mistakes in the generation of this code. Teachers will have over a week to collect questionnaire data in order to minimise loss of data through student absences from school.

Class randomisation to condition will take place after pre-test, at the level of school.

#### Stage 2; the intervention

Participants are taught within their usual classes. Each class is facilitated by 2-4 staff including a lead who will be a NHS mental health specialist, and a teaching assistant. Facilitators in the education and contact condition include at least one facilitator who has lived experience of mental illness. As well as the facilitators a minimum of one teacher from the school will be present in each class. Fidelity of the intervention delivery between conditions will be assessed with a pre-developed checklist on the intervention day. One class per condition, per school will be assessed for fidelity by K.C.

#### Stage 3; post-tests

Approximately 2 weeks after the intervention day post-intervention questionnaires will be completed by participants, and then again at 6 month follow up.

### Primary outcome measure

#### Stigma of mental illness

The Reported & Intended Behaviour Scale (RIBS) [65] assesses behaviour related to the stigma of mental illness. The RIBS takes around 1-2 minutes to complete and rates participants' willingness to have contact with individuals who are experiencing mental illness (e.g. 'In the future I would be willing to live with someone with a mental health problem'), as well as their current and past experiences (e.g. 'Are you currently living with, or have you ever lived with, someone with a mental health problem?'), though only the former questions generates the participants final score. The RIBS has a test-retest reliability of 0.75, and Cronbach's alpha for items 5-8 (those which generate the participants final score) is 0.85.

### Secondary outcome measures

#### Knowledge of mental illness

Knowledge of mental illness will be assessed in two ways. The Mental Health Knowledge Scale (MAKS) [66] assesses six areas of stigma-related knowledge: help-seeking, recognition, support, employment, treatment, and recovery, takes 1-2 minutes to complete and has a test-retest reliability of 0.71.

Two vignettes will be employed to assess knowledge of mental illness developed by Jorm et al. [67].

#### Emotional well-being

The Strengths and Difficulties Scale (SDQ) [68] will be used to assess the emotional well-being and consists of 25 items which generate scores along five subscales: conduct problems, hyperactivity-inattention, emotional symptoms, peer problems, and pro-social behaviour, as well as producing a total difficulties score. The SDQ has been validated for use with adolescents age 11-16 with a Cronbach's alpha of 0.82 for the total difficulties scale [68].

#### Resilience

Resilience will be measured using a 15 item [69] version of Wagnild and Young's [70] Resilience Scale (Cronbach's alpha of between 0.72-0.94) [71] and concurrent validity is supported by correlations between the Resilience scale and measures of depression, morale, and life satisfaction. The Resilience scale has been used previously in three studies with adolescents [72-74].

#### Help-seeking

Attitudes to help-seeking will be assessed by responses to the question 'In the next 12 months if you were to experience a mental illness, how likely are you to seek help?'

### Exploratory outcome measure

#### Schizotypal thinking

The Schizotypal Personality-Brief Form (SPQ) [75,76] assesses three subscales relating to schizotypal thinking; cognitive-perceptual deficits, interpersonal deficits, and disorganisation. It takes approximately 2 minutes to complete and has an internal reliability of 0.76, a test-retest reliability of 0.90, and has been validated for use with adolescents [77].

### Power and sample size calculation

Assuming an intra-cluster correlation coefficient (ICC) of 0.037 [78] (Aberdeen University: Health Services Research Unit) and a cluster size of approximately 30 students per class, 738 participants would be needed to detect a 0.3 effect size, or 1658 participants to detect a 0.2 effect size. Depending on the size of the included schools, the proposed research will allow for detection of an effect size between 0.3-0.2.

### Analysis

Data will be analysed applying appropriate general linear models. In order that the clustering of the sample does not bias the analysis pre-test outcome measures will be entered as a covariate. Subgroup analyses will look at gender, ethnicity, religion, and mental health status of self, family, and friends in relation to the hypotheses and data analysed using appropriate general linear models. Intention to treat analyses will be used.

## Discussion

The strengths of the proposed research are both in its potential for increasing efficacy by the use of contact combined with education to combat primarily stigma, and also in improving mental health literacy and resilience/well-being. Previous research suggests that the intervention may have some effect on stigma even at 6-month follow up, due to this influence of contact combined with education. Pinfold et al. [79] for example, employed contact with an individual with personal experience of living with mental illness in their intervention with 14-15 year old students. Although their intervention was brief, lasting for just two one-hour workshops, a significant positive change in stigmatising attitudes was found both immediately following the intervention and also at 6-month follow up.

Previous interventions have tended to focus on one or at most two of the concepts of stigma, mental health literacy, and resilience/well-being, rather than all three [35-40,42]. This is despite evidence inferring that the three concepts are to some extent interlinked [20,21]. For example, Naylor et al.'s [41] intervention significantly reduced conduct problems and improved prosocial behaviour, despite the fact that the intervention did not contain any features designed to reduce mental illness or promote mental health. Though the research is not directly designed to look at this, if stigma, mental health literacy, and resilience/well-being are interlinked, it may be that by addressing all three simultaneously, a greater impact can be achieved.

The current intervention also addresses many of the methodological issues which have been highlighted by previous systematic research reviews as problematic [9,48]. The study has a high level of power; utilises an active control group; measures and evaluates demographic differences including ethnicity, religion, and gender for response to the intervention; attempts to improve consent rate by utilising an opt-out consent method; is delivered by the research team rather than by teachers from the individual schools and assessed for fidelity of implementation. The research measures mediator variables such as resilience as well as baseline characteristics of the individual such as whether they know someone with a mental illness, and the exact nature and content of the intervention will be explicitly described and freely available.

If positive trends result from the present feasibility trial this will support the development of further longitudinal work on a larger scale, something which is urgently needed in this field.

## Competing interests

The authors declare that they have no competing interests.

## Authors' contributions

KC is chief investigator on the project and drafted the manuscript. KC, PP, and ET contributed to the development and implementation of the intervention. MB, PP, and ET supervise KC. All authors contributed to the design of the study. All authors contributed to the editing of the manuscript and have read and approved the final manuscript.

## Pre-publication history

The pre-publication history for this paper can be accessed here:

http://www.biomedcentral.com/1471-244X/12/23/prepub

## References

[B1] McCrone P Dhanasiri S Patel A Knapp M Lawton-Smith S Paying the price: the cost of MH care in England to 2026 http://www.kingsfund.org.uk/publications

[B2] Department of Health The mental health policy implementation guide 2001 London: Department of Health

[B3] Department of Health Making it happen: a guide to developing mental health promotion 2004 http://www.publications.doh.gov.uk/pdfs/makingithappen.pdf

[B4] Sainsbury Centre for Mental Health Policy Paper: The future of mental health, a vision for 2015 2006 London: SCMH http://www.centreformentalhealth.org.uk/pdfs/mental_health_futures_policy_paper.pdf

[B5] Office of the Deputy Prime Minister Mental Health and Social Exclusion: The social exclusion unit report 2004 London: HMSO

[B6] The Royal College of Psychiatrists Mental illness and stigma, Module 217 Office for National Statistics 1998

[B7] Her Majesty's Government. Children Act The Stationery Office 2004

[B8] The Office for National Statistics Omnibus survey 2008 ONS

[B9] Spence SH Shortt AL Research review: can we justify the widespread dissemination of universal, school-based interventions for the prevention of depression among children and adolescents? J Child Psychol Psychiatry 2007 48 526 542 10.1111/j.1469-7610.2007.01738.x 17537069

[B10] Merry S McDowell H Hetrick S Bir J Muller N Psychological and/or educational interventions for the prevention of depression in children and adolescents (Cochrane review) The Cochrane Library 2007 2 Chichester, UK: John Wiley and Sons, Ltd 10.1002/14651858.CD003380.pub2 14974014

[B11] Wells J Barlow J Stewart-Brown S A systematic review of the universal approaches to MH promotion in schools Heal Educ 2003 103 4 197 220 10.1108/09654280310485546

[B12] Kessler RC Amminger GP Aguilar-Gaxiola S Alonso J Lee S Ustun TB Age of onset of mental disorders: a review of recent literature Curr Opin Psychiatr 2007 20 359 364 10.1097/YCO.0b013e32816ebc8c PMC1925038 17551351

[B13] Olsson DP Kennedy MG Mental health literacy among young people in a small US town: recognition of disorders and hypothetical helping responses Early Interv Psychiatry 2010 4 291 298 10.1111/j.1751-7893.2010.00196.x 20977685

[B14] Morgan AJ Jorm AF Recall of news stories about mental illness by Australian youth: associations with help-seeking attitudes and stigma Aust NZ J Psychiat 2009 43 866 872 10.1080/00048670903107567 19670060

[B15] Steinberg LD Cognitive and affective development in adolescence Trends Cogn Sci 2005 9 69 74 10.1016/j.tics.2004.12.005 15668099

[B16] McGorry PD Mcfarlane C Patton GC Bell R Hibbert ME Jackson HJ Bowes G The prevalence of prodromal features of schizophrenia in adolescence - a preliminary survey Acta Psychiatr Scand 1995 92 4 241 249 10.1111/j.1600-0447.1995.tb09577.x 8848947

[B17] Raine A Schizotypal personality: neurodevelopmental and psychosocial trajectories Ann Rev Clin Psychol 2006 2 291 326 10.1146/annurev.clinpsy.2.022305.095318 17716072

[B18] Mental Health Foundation Respect costs nothing: A survey of discrimination face by people with experience of mental illness in Aotearoa New Zealand 2004 Auckland: Mental Health Foundation

[B19] LikeMinds The power of contact; Project to Counter Stigma and Discrimination Associated with Mental Illness 2005 http://www.likeminds.org.nz/file/downloads/pdf/1power-of-contact.pdf

[B20] Schomerus G Matschinger H Angermeyer MC The stigma of psychiatric treatment and help-seeking intentions for depression Eur Archives Psychiat Clin Neurosci 2009 259 5 298 306 10.1007/s00406-009-0870-y 19224105

[B21] Kelly CM Jorm AF Wright A Improving MH literacy as a strategy to facilitate early intervention for mental disorders Med J Aust 2007 187 7 s26 s30 17908021 10.5694/j.1326-5377.2007.tb01332.x

[B22] Kessler RC Keller MB Wittchen HU The epidemiology of generalized anxiety disorder Psychiatr Clin North Am 2001 24 19 39 10.1016/S0193-953X(05)70204-5 11225507

[B23] Clark DB Jones BL Wood DS Substance use disorder trajectory classes: Diachronic integration of onset age, severity, and course Addict Behav 2006 31 6 995 1009 10.1016/j.addbeh.2006.03.016 16675151

[B24] Nierenberg AA Quitkin FM Kremer C Placebo-controlled continuation treatment with mirtazapine: acute pattern of response predicts relapse Neuropsychopharmacology 2004 29 5 1012 1018 10.1038/sj.npp.1300405 14997172

[B25] Kidger J Donovan JL Biddle L Campbell R Gunnell D Supporting adolescent emotional health in schools: a mixed methods study of student and staff views in England BMC Public Health 2009 9 403 10.1186/1471-2458-9-403 19878601 PMC2777165

[B26] Woolfson R Woolfson L Mooney L Bryce D Young people's views of mental health education in secondary schools: a Scottish study Child: care, health, and development 2008 35 6 790 798 10.1111/j.1365-2214.2008.00901.x 18991977

[B27] Hooven C Herting JR Snedker KA Long-term outcomes for the promoting CARE suicide prevention program Am J Health Behav 2010 34 721 736 20604697 10.5993/ajhb.34.6.8 PMC3119363

[B28] Reynolds AJ Temple JA Ou SR Robertson DL Mersky JP Topitzes JW Niles MD Effects of a school-based, early childhood intervention on adult health and well-being - A 19 year follow-up of low income families Arch Pediatr Adolesc Med 2007 161 8 730 739 10.1001/archpedi.161.8.730 17679653

[B29] Mercy JA Saul J Creating a healthier future through early interventions for children JAMA-J Am Med Assoc 2009 301 21 2262 2264 10.1001/jama.2009.803 19491188

[B30] Healthy Schools: Department for education and Department of Health http://www.uclan.ac.uk/schools/school_of_health/research_projects/hsu/files/national_healthy_schools_status_guide.pdf

[B31] Clarke GN Hawkins W Murphy M Sheeber L School-based primary prevention of depressive symptomatology in adolescents: findings from two studies J Adolesc Res 1993 8 183 204 10.1177/074355489382004

[B32] Pattison C Lynd-Stevenson RM The prevention of depressive symptoms in children: the immediate and long-term outcomes of a school-based program Behaviour Change 2001 18 92 102 10.1375/bech.18.2.92

[B33] Quayle D Dziurawiec S Roberts C Kane R Ebsworthy G The effect of an optimism and lifeskills program on depressive symptoms in preadolescence Behav Chang 2001 18 194 203 10.1375/bech.18.4.194

[B34] Shochet IM Dadds MR Holland D Whitefield K Harnett PH Osgarby SM The efficacy of a universal school-based program to prevent adolescent depression J Clin Child Psychol 2001 30 303 315 10.1207/S15374424JCCP3003_3 11501248

[B35] Spence SH Sheffield J Donovan CL Long-term outcome of a school-based universal approach to prevention of depression in adolescents J Consult Clin Psychol 2005 73 160 167 15709843 10.1037/0022-006X.73.1.160

[B36] Merry S McDowell H Wild CJ Bir J Cunliffe R A randomised placebo controlled trial of a school-based depression prevention program J Am Acad Child Adolesc Psychiat 2004 43 538 547 10.1097/00004583-200405000-00007 15100560

[B37] Harnett PH Dadds MR Training school personnel to implement a universal school-based prevention of depression program under real-world conditions J Sch Psychol 2004 42 343 357 10.1016/j.jsp.2004.06.004

[B38] Pössel P Horn AB Groen G Hautzinger M School-based prevention of depressive symptoms in adolescents: A 6-month follow-up J Am Acad Child Adolesc Psychiat 2004 43 1003 1010 10.1097/01.chi.0000126975.56955.98 15266195

[B39] Pössel P Baldus C Horn AB Groen G Hautzinger M Influence of general self-efficacy on the effects of a school-based universal primary prevention program of depressive symptoms in adolescents: A randomized and controlled follow-up study J Child Psychol Psychiatry 2005 46 982 994 10.1111/j.1469-7610.2004.00395.x 16109001

[B40] Sheffield JK Spence SH Rapee RM Kowalenko N Wignall A Davis A Evaluation of universal, indicated, and combined cognitive-behavioural approaches to the prevention of depression among adolescents J Consult Clin Psychol 2006 74 66 79 16551144 10.1037/0022-006X.74.1.66

[B41] Naylor PB Cowie HC Walters SJ Talamelli L Dawkins J Impact of a mental health teaching programme on adolescents Br J Psychiatry 2009 194 365 370 10.1192/bjp.bp.108.053058 19336791

[B42] Roberts G Somers J Dawe J Passy R Mays C Carr G Shiers D Smith J On the edge: a drama-based mental health education programme on early psychosis for schools Early Interven Psychiat 2007 1 168 176 10.1111/j.1751-7893.2007.00025.x

[B43] Burns EJ Walrath C Glass-Siegel M School-based mental health in Baltimore Behav Modif 2004 28 491 512 10.1177/0145445503259524 15186512

[B44] Scott TM A school-wide example of positive behavioural support J Posit Behav Interv 2001 3 88 94 10.1177/109830070100300205

[B45] Stormshak B Dishion T Light J Implementing family-centered interventions within the public middle school: linking service delivery to change in student problem behaviour J Abnorm Child Psychol 2005 33 723 733 10.1007/s10802-005-7650-6 16328747

[B46] beyondblue Schools Research Initiative: Report of Key Findings (2003-2005) http://www.beyondblue.org.au Retrieved May 2010.

[B47] Sawyer MG Pfeiffer S Spence SH Bond L Graetz B Kay D Patton G Sheffield J School-based prevention of depression: a randomised controlled study of the beyondblue schools research initiative J Child Psychol Psychiatry 2010 51 2 199 209 10.1111/j.1469-7610.2009.02136.x 19702662

[B48] Schachter HM Girardi A Ly M Lacroix D Lumb AB van Berkom J Gill R Effects of school-based interventions on MH stigmatization: a systematic review Child and Adolesc Psychiat MH 2008 2 1 18 10.1186/1753-2000-2-18 PMC2515285 18644150

[B49] Flay BR Biglan A Boruch RF Castro FG Gottfredson D Kellam SG Standards of evidence: criteria for efficacy, effectiveness and dissemination Prev Sci 2005 6 151 175 10.1007/s11121-005-5553-y 16365954

[B50] Weare K Markham W What do we know about promoting MH through schools? IUHPE: Promotion and Education 2005 XII 3-4 14 18 10.1177/10253823050120030104 16739496

[B51] Lister Sharpe D Chapman S Stewart Brown S Sowden A Health promoting schools and health promotion in schools: two systematic reviews Health Technol Assess 1999 3 22 10683593

[B52] Hoagwood KE Olin SS Kerker BD Kratochwill TR Crowe M Saka N Empirically based school interventions targeted at academic and mental health functioning J Emot Behav Dis 2007 15 2 66 92 10.1177/10634266070150020301

[B53] Pinfold V Stuart H Thornicroft G Arbolelda-Florez J Working with young people: the impact of mental health awareness programs in schools in the UK and Canada World Psychiatry 2005 4 suppl. 1 48 52

[B54] Children and Adolescent's MH Coalition Children and adolescents' mental health: the policy, the progress made, the challenges 2010 http://www.mentalhealth.org.uk/campaigns/children-and-young-people-coalition/

[B55] Corrigan PC River LP Lundin RK Penn DL Uphoff W Campion J Mathisen J Gagnon C Bergman M Goldstein H Kubiak MA Three strategies for changing attributions about severe mental illness Schizophrenia Bull 2001 27 2 187 195 10.1093/oxfordjournals.schbul.a006865 11354586

[B56] Rusch N Angermeyer MC Corrigan PW Mental illness stigma: concepts, consequences, and initiatives to reduce stigma Eur Psychiat 2005 20 529 539 10.1016/j.eurpsy.2005.04.004 16171984

[B57] Allport GW The nature of prejudice 1954 Reading, MA: Addison-Wesley

[B58] Couture SM Penn DL Interpersonal contact and the stigma of mental illness: a review of the literature J Ment Heal 2003 12 291 305 10.1080/09638231000118276

[B59] Fossati A Raine A Borroni S Maffei C Taxonic structure of schizotypal personality in nonclinical subjects: Issues of replicability and age consistency Psychiat Res 2007 152 2-3 103 112 10.1016/j.psychres.2004.04.019 17434601

[B60] Altman DG Better reporting of randomised controlled trials: the CONSORT statement Brit Med J 1996 313 570 571 10.1136/bmj.313.7057.570 8806240 PMC2352018

[B61] Altman DG Schulz KF Moher D Egger M Davidoff F Elbourne D The revised CONSORT statement for reporting randomized trials: explanation and elaboration Ann Intern Med 2001 134 8 663 694 11304107 10.7326/0003-4819-134-8-200104170-00012

[B62] O' Reilly G A CBT Workbook for Children and Adolescents. School of Psychology 2004 University College Dublin http://www.juvenilementalhealthmatters.com/CBT_Workbook.html

[B63] Changes, Staffordshire Changes YP mental health programme http://www.changes.org.uk/html/young_people.html

[B64] Rosaria M Galanti MR Siliquini R Cuomo L Melero JC Panella M Faggiano F Testing anonymous link procedures for follow-up of adolescents in a school-based trial: the EU-DAP pilot study Prev Med 2007 44 174 177 10.1016/j.ypmed.2006.07.019 16979751

[B65] Evans-Lacko S Rose D Little K Development and psychometric properties of the reported and intended behaviour scale (RIBS): a stigma-related behaviour measure Epidemiol Psychiatr Sci 2011 20 3 263 271 10.1017/S2045796011000308 21922969

[B66] Evans-Lacko S Little K Meltzer H Rose D Rhydderch D Henderson C Thornicroft G Development and psychometric properties of the mental health knowledge schedule (MAKS) Can J Psychiat 2010 55 7 440 448 10.1177/070674371005500707 20704771

[B67] Jorm AF Korten AE Jacomb PA "Mental health literacy": a survey of the public's ability to recognise mental disorders and their beliefs about the effectiveness of treatment Med J Aust 1997 166 4 182 186 9066546 10.5694/j.1326-5377.1997.tb140071.x

[B68] Goodman R Meltzer H Bailey V The strengths and difficulties questionnaire: A pilot study on the validity of the self-report version Eur Child Adolesc Psychiat 1998 7 3 125 130 10.1007/s007870050057 9826298

[B69] Neill JT Dias KL Adventure Education and Resilience - The Double-Edged Sword Home J Adven Educ Outdoor Leadership 2001 1 2 35 42 10.1080/14729670185200061

[B70] Wagnild GM Young HM Development and psychometric evaluation of the resilience scale J Nurs Meas 1993 1 165 178 7850498

[B71] Wagnild G A review of the Resilience Scale J Nurs Meas 2009 17 2 105 113 10.1891/1061-3749.17.2.105 19711709

[B72] Black C Ford-Gilboe M Adolescent mothers: Resilience, family health work and health-promoting practices J Adv Nurs 2004 48 4 351 360 10.1111/j.1365-2648.2004.03204.x 15500529

[B73] Rew L Taylor-Seehafer M Thomas NY Yockey RD Correlates of resilience in homeless adolescents J Nurs Sch 2001 33 1 33 40 10.1111/j.1547-5069.2001.00033.x 11253578

[B74] Hunter AJ Chandler GE Adolescent resilience J Nurs Sch 1999 31 2 243 247 10.1111/j.1547-5069.1999.tb00488.x 10528454

[B75] Raine A Benishay D The SPQ-B A brief screening instrument for schizotypal personality disorder J Personal Disord 1995 9 4 346 355 10.1521/pedi.1995.9.4.346

[B76] Raine A The SPQ A scale for the assessment of schizotypal personality based on DSM-III-R criteria Schizophr Bull 1991 17 555 564 1805349 10.1093/schbul/17.4.555

[B77] Fonseca-Pedrero E Paino-Pineiro M Lemos-Giraldez S Villazon-Garcia U Muniz J Validation of the Schizotypal Personality Questionnaire-Brief Form in adolescents Schizophr Res 2009 111 1-3 53 60 10.1016/j.schres.2009.03.006 19342199

[B78] Database of intra-correlation coefficients (ICCs) Aberdeen University: Health Services Research Unit http://www.abdn.ac.uk/hsru/research/research-tools/study-design

[B79] Pinfold V Toulmin H Thornicroft G Huxley P Farmer P Graham T Reducing psychiatric stigma and discrimination: evaluation of educational interventions in UK secondary schools Br J Psychiatry 2003 182 342 346 10.1192/bjp.182.4.342 12668411

